# Tensor Algebra-based Geometrical (3D) Biomacro-Molecular Descriptors for Protein Research: Theory, Applications and Comparison with other Methods

**DOI:** 10.1038/s41598-019-47858-2

**Published:** 2019-08-06

**Authors:** Julio E. Terán, Yovani Marrero-Ponce, Ernesto Contreras-Torres, César R. García-Jacas, Ricardo Vivas-Reyes, Enrique Terán, F. Javier Torres

**Affiliations:** 1 Universidad San Francisco de Quito (USFQ), Grupo de Medicina Molecular y Translacional (MeM&T), Colegio de Ciencias de la Salud (COCSA), Escuela de Medicina, Edificio de Especialidades Médicas, Quito, Pichincha Ecuador; 2 Universidad San Francisco de Quito (USFQ), Grupo de Química Computacional y Teórica (QCT-USFQ), Departamento de Ingeniería Química, and Instituto de Simulación Computacional (ISC-USFQ), Quito, Pichincha Ecuador; 3 Universidad de San Buenaventura - Cartagena - Facultad de Ciencias de la Salud - Grupo de Investigación Microbiología & Ambiente (GIMA) - Calle Real de Ternera, Diagonal 32, No. 30-966, Cartagena, Código postal: 1300 10 Colombia; 4 Cátedras CONACYT - Departamento de Ciencia de la Computación, Centro de Investigación Científica y de Educación Superior de Ensenada (CICESE), Ensenada, Baja California Mexico; 5 Grupo de Química Cuántica y Teórica de la Universidad de Cartagena-Facultad de Ciencias Exactas y Naturales. Programa de Química. Campus de San Pablo and Grupo GINUMED Corporacion Universitaria Rafal Nuñez. Facultad de Salud. Programa de Medicina., Cartagena, Colombia; 6 Grupo CipTec, Facultad de Ingenierias. Fundacion Universitaria Tecnologico Comfenalco – Cartagena, Cartagena, Bolívar Colombia

**Keywords:** Computational models, Protein function predictions

## Abstract

In this report, a new type of tridimensional (3D) biomacro-molecular descriptors for proteins are proposed. These descriptors make use of multi-linear algebra concepts based on the application of 3-linear forms (*i*.*e*., Canonical Trilinear (Tr), Trilinear Cubic (TrC), Trilinear-Quadratic-Bilinear (TrQB) and so on) as a specific case of the *N*-linear algebraic forms. The definition of the k^th^ 3-tuple similarity-dissimilarity spatial matrices (*Tensor’s Form*) are used for the transformation and for the representation of the existing chemical information available in the relationships between three amino acids of a protein. Several metrics (*Minkowski-type, wave-edge*, etc) and multi-metrics (*Triangle area, Bond-angle*, etc) are proposed for the interaction information extraction, as well as probabilistic transformations (*e*.*g*., simple stochastic and mutual probability) to achieve matrix normalization. A generalized procedure considering amino acid level-based indices that can be fused together by using aggregator operators for descriptors calculations is proposed. The obtained results demonstrated that the new proposed 3D biomacro-molecular indices perform better than other approaches in the SCOP-based discrimination and the prediction of folding rate of proteins by using simple linear parametrical models. It can be concluded that the proposed method allows the definition of 3D biomacro-molecular descriptors that contain orthogonal information capable of providing better models for applications in protein science.

## Introduction

It is well accepted that geometrical representations of chemical structures contain not only descriptive information but insights of the native configuration of the represented molecules. In the case of proteins, it has been observed that their tridimensional (3D) structure provides information about their function in living organisms^1^. Using graphic approaches to study biological and medical systems can provide an intuitive vision and useful insights for helping analyze complicated relations therein, as indicated by many previous studies on a series of important biological topics (particularly for the topics of enzyme kinetics^2–5^, protein folding rates^6–9^, and low-frequency internal motion^10,11^).

Thus, the use of 3D molecular descriptors (MDs) can be considered as an approach for inferring information about structural properties and their related quantities. A good number of prediction models that link 3D chemical structures with activity or properties (QSAR/QSPR) have been generated from 3D-MDs, which have been extensively used for the characterization of organic molecules and small chemical systems^12^. However, in the case of proteins a few biomacro-molecular indices have been proposed for sequence codification and spatial information extraction^13–15^. This indicates that the approaches based on MDs have not been completely exploited, and it could be considered a field subjected to further theoretical development in protein science.

The modelling of physicochemical properties and biological interactions for proteins require the extraction of information regarding sequence, spatial configuration and the chemical characteristics of every amino acid present on the structure^12,16–18^. Thus, it is important to generate new 3D-MDs for proteins that consider all these features present in 3D structures that provide new, non-redundant information and a more complete characterization of them.

Marrero-Ponce *et al*. introduced a new set of MDs that consider topology (2D) related characteristics for organic molecules^19–23^, which has been included in QuBiLs MAS (Quadratic, Bilinear and N-Linear Maps based on graph-theoretic electronic-density Matrices and Atomic Weightings) software^24^. These 0-2D and chiral MDs were obtained codifying the structural information, using algebraic bilinear forms, and considering electronic density graph-based matrices. Based on their performance and seeking a generalization of this mathematical proposal (N-linear algebraic forms, related to tensor algebra), the definition of geometrical 3D-MDs for organic molecules was also proposed. This approach allowed the use of N-linear algebraic forms as well as other mathematical considerations such as metrics and aggregation operators to increase the information extraction for the resultant indices^25–27^. The aforementioned approach was named QuBiLs MIDAS (Quadratic, Bilinear and N-Linear Maps based on N-tuple Spatial Metric [(Dis)-Similarity] Matrices and Atomic Weightings)^28^ and several preliminary studies with the QuBiLS-MIDAS 3D-MDs demonstrated a satisfactory behavior, suggesting that this algebraic strategy yields information-rich indices of relevance in chemoinformatic studies^26^.

There are several applications in protein science such as the prediction of protein structural classes^29^ and the folding rate of proteins^30^, which have defined benchmark data sets that have been used in numerous articles^31–34^. It has been observed that the amino acid sequence and the various interactions between every amino acid present on a protein, could give information concerning the global stability of the native structure and folding process, indicating that the folding rate of proteins do not consider solely thermodynamic factors^35^. Therefore, the folding rate of proteins could provide information about the function of a protein based on its geometrical and topological configurations^36,37^.

Regarding structural class prediction, it has been used as a tool to predict protein function and evolution since the 1970s^38^. Based on the importance and amount of information related to these two properties, several computational methodologies have been proposed for their calculation. Considering the case of structural classification, there are several methods proposed for this purpose: the amino acid composition (AAC)^33^, pair-coupled amino acid composition^39^, pseudo amino acid composition (PseAAC)^14^, and a mathematical based strategy considering bilinear descriptors^40^. Concerning protein folding rate, there are several indices that consider the topology/geometry of proteins and the number of contacts between amino acids for the prediction of this property^15,30,41–43^.

The major disadvantage of the AAC-based methods is the reduced consideration of the interaction effects generated by the sequence of the protein, generating lower quality on the prediction. There have been several approaches based on PseAAC that were proposed to improve the prediction of these type of descriptors^44–49^. Regarding the descriptors generated for protein folding, they consider geometrical/topological concepts, distance between the residues in contact as well as long- and short-range interactions based on the conformation of the protein. However, the disadvantage of these approaches is that they do not consider the whole 3D nature of proteins and the information contained on it, since it has been proven that folding rate does not only depend on sequence^36^.

As demonstrated by a series of recent publications^50–55^ and summarized in a comprehensive review^56^, to develop a really useful predictor for a biological system, it can be recommended to follow Chou’s 5-step rule which contains the following steps: (a) select or construct a valid benchmark dataset to train and test the predictor; (b) represent the samples with an effective formulation that can truly reflect their intrinsic correlation with the target to be predicted; (c) introduce or develop a powerful algorithm to conduct the prediction; (d) properly perform cross-validation tests to objectively evaluate the anticipated prediction accuracy; (e) establish a user-friendly web-server for the predictor that is accessible to the public. Papers presented for developing a new sequence-analyzing method or statistical predictor by observing the guidelines of Chou’s 5-step rules have the following notable merits: (1) crystal clear in logic development, (2) completely transparent in operation, (3) easily to repeat the reported results by other investigators, (4) with high potential in stimulating other sequence-analyzing methods, and (5) very convenient to be used by the majority of experimental scientists.

The main aim of this study is the introduction of a new class of 3D protein MDs based on N-linear algebraic forms that consider several mathematical tools as concept generalization for enhanced information extraction from proteins. The utility of these novel 3D-biomacro-molecular indices will be evaluated by the prediction of SCOP-structural classes of proteins and its folding rate by using Linear Discriminant Analysis (LDA) and Multiple Linear Regression (MLR) techniques, respectively.

## Theoretical Framework

The concept of algebraic based (bilinear) 3D-MDs was proposed in 2015 by Marrero- Ponce *et al*. as a tool for protein structural codification^40^, and an initial extension of a geometric distance matrix^12,57^ for a protein was obtained.

However, the use of tensor algebra to codify relations between more than 2 atoms (3 and 4 atoms) has been used for organic molecules as a strategy for obtaining more information from the geometrical 3D molecular structure^26^. In this work, the N-tuple algebraic form concept (N = 3) will be evaluated for the calculation of 3D-protein descriptors.

### Definitions for the total and amino acid level 3D protein descriptors based on three-linear forms

The definition for any *k*^*th*^ three-linear biomacro-molecular descriptors for a protein must consider a canonical basis set and the application of N-linear forms (maps) in a ${{\mathbb{R}}}^{n}$ space; Eq. (1) indicates the mathematical expression for this definition:

1 $${}_{tr}{}^{k}L=t{r}^{k}(\bar{x},\bar{y},\bar{p})=\sum _{i=1}^{n}\,\sum _{j=1}^{n}\,\sum _{l=1}^{n}\,{z}_{ijl}^{k}{x}^{i}{y}^{j}{p}^{l}$$

This trilinear form could be defined by using matrices as follows,

2 $${}_{{\boldsymbol{tr}}}{}^{{\boldsymbol{k}}}{\boldsymbol{L}}=[{\boldsymbol{X}}]\,{{\mathbb{Z}}}^{{\boldsymbol{k}}}{[{\boldsymbol{Y}}]}^{{\boldsymbol{T}}}{[{\boldsymbol{P}}]}^{{\boldsymbol{T}}}={X}_{(1\times {\bf{n}}\times 1)}\,{{\mathbb{Z}}}_{({\bf{n}}\times {\bf{n}}\times {\bf{n}})}^{{\boldsymbol{k}}}{Y}_{({\bf{n}}\times 1\times 1)}{P}_{(1\times 1\times {\bf{n}})}$$

where, ${}_{tr}{}^{k}L\,$ is the resulting trilinear form MD, *n* is the number of amino acids (*aa*) present on the protein, $[X],\,[Y],\,[P]$ are the macro-molecular vectors containing *x*^1^,*…*, *x*^*n*^, *y*^1^*,…,y*^*n*^ and *p*^1^,*…*, *p*^*n*^ elements, which are the physicochemical properties of every *aa* present in the protein structure^58,59^. A Table indicating all physicochemical properties considered on this study is available on the Supplementary Material SMI-A. The *k*^*th*^ *total three-tuple-(dis)similarity matrices* (T-TDSM) (${{\mathbb{Z}}}^{k}$) is a three-order tensor whose elements ${z}_{ijl}^{k}$ are calculated by using relationships (multi-metrics) between three *aa*. These relationships will be discussed in Section **2**.**4**.

**Figure 1:**
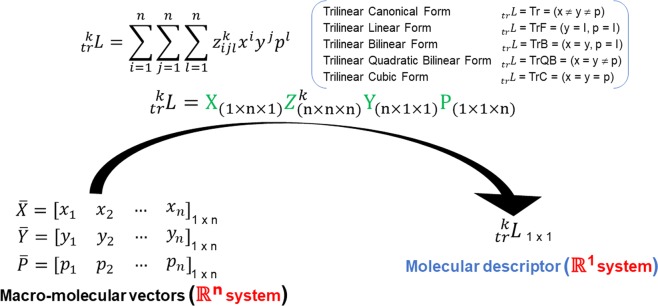
Schematic indication of the transformation of the information contained on macro-molecular vectors using spatial information of the protein (Three-Tuple-(Dis)Similarity-Matrices) (TDSM) and algebraic forms. Where *n* is the number of amino acids present on the protein, [*X*], [*Y*], [*P*] are macro-molecular vectors; *z*^*k*^_*ijl*_ are elements of the TDSM and _*tr*_*L* is the resulting MD. These algebraic forms are defined by the physicochemical nature of the macro-molecular vectors.

**Figure 2:**
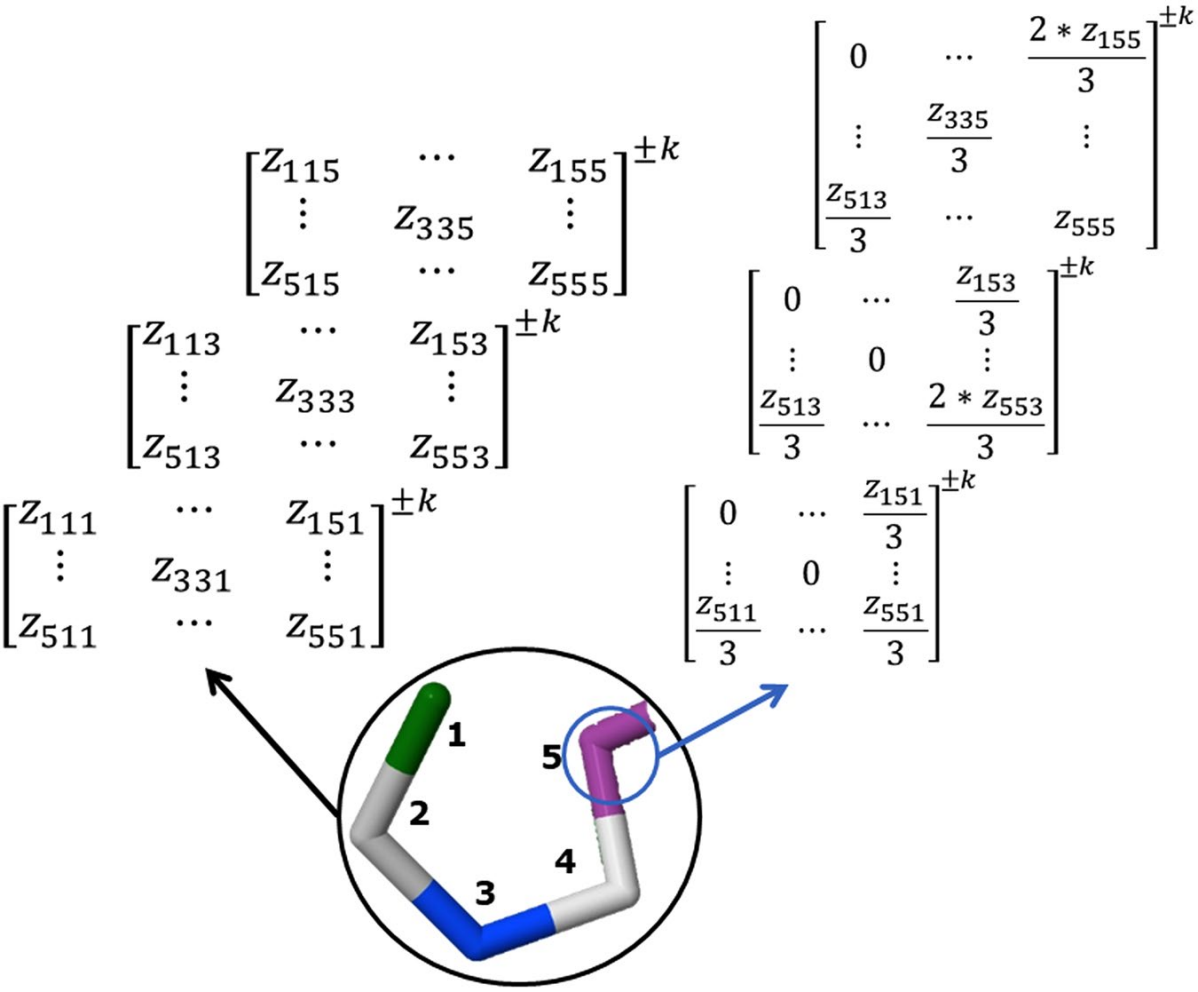
Graphical representation of the differences on the computation between (**a**) total and (**b**) amino acid-based tensors for the novel 3D algebraic MDs for a simple example, *i*.*e*., truncated peptide PDB file (5WRX).

Based on the physicochemical nature of the properties used for the macromolecular vectors conformation, the following algebraic forms could be defined: (1) Trilinear Canonical (when all macro-molecular vectors are configured differently, that is, using 3 different *aa* properties) (see Fig. 1), (2) Trilinear linear (when 2 of the macro-molecular vectors are the identity vector and the other one is an *aa* property), (3) Trilinear bilinear (when 2 macro-molecular vectors have the same configuration (that is to say, by using the same aa property) and the other one is the identity vector), (4) Trilinear quadratic bilinear (when 2 macro-molecular vectors have the same configuration and the other one has a different aa property from the previous), and (5) Trilinear cubic (when all the macro-molecular vectors have the same configuration, *i*.*e*., use the same aa property).

Moreover, the definition of *aa*-based *k*^*th*^ three-linear MDs for every *aa* in the protein is shown in Eq. (3):

3 $${}_{tr}{}^{k}L_{aa}=t{r}^{aa,k}(\bar{x}\,,\,\bar{y},\bar{p})=\sum _{i=1}^{n}\,\sum _{j=1}^{n}\,\sum _{l=1}^{n}\,{z}_{ijl}^{aa,k}{x}^{i}{y}^{j}{p}^{l}=[X]\,{{\mathbb{Z}}}^{aa,k}{[Y]}^{T}{[P]}^{T}\,\forall \,aa=1,2,\ldots ,n$$

where, *x*^1^,*…*, *x*^*n*^, *y*^1^,*…*, *y*^*n*^ and *p*^1^,*…*, *p*^*n*^ are the components of the macro-molecular vectors.

The *k*^*th*^ *amino acid-level three-tuple-(dis)similarity matrices* (A-TDSM) (${{\mathbb{Z}}}^{aa,k}$) with elements ${z}_{ijl}^{aa,k}$ are computed by considering the following rules:

4 $$\begin{array}{ll}{z}_{ijl}^{aa,k}={z}_{ijl}^{k} & {\rm{if}}\,{\boldsymbol{i}}\wedge {\boldsymbol{j}}\wedge {\boldsymbol{l}}={\bf{a}}{\bf{a}}\\ {z}_{ijl}^{aa,k}=\frac{2}{3}{z}_{ijl}^{k} & {\rm{if}}\,{\boldsymbol{i}},{\boldsymbol{j}}\vee {\boldsymbol{j}},{\boldsymbol{l}}\vee {\boldsymbol{i}},{\boldsymbol{j}}={\bf{a}}{\bf{a}}\\ {z}_{ijl}^{aa,k}=\frac{1}{3}{z}_{ijl}^{k} & {\rm{if}}\,{\boldsymbol{i}}\,\vee {\boldsymbol{j}}\vee {\boldsymbol{l}}={\bf{a}}{\bf{a}}\\ {z}_{ijl}^{aa,k}=0 & {\rm{otherwise}}\end{array}$$

**Figure 3:**
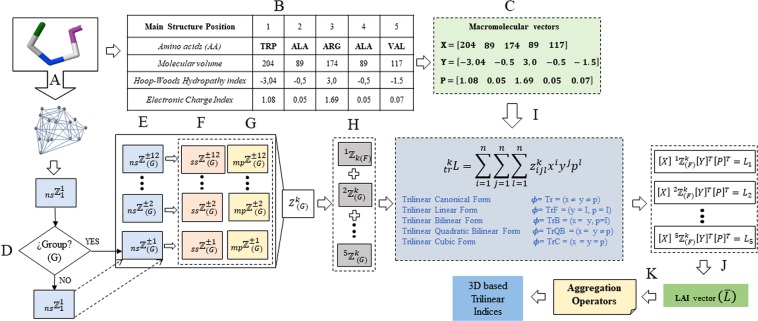
Novel molecular descriptors calculation illustration. (**A**) Protein structure is filtered considering a protein representation (Section 2.4.) for calculating the relationship between two (metrics, SMI-C) and three amino acids (multi-metrics, Table 2). (**B**,**C**) The computation of the macromolecular vectors considers a group of physicochemical properties and the sequence of the structure (Section 2.1.). (**D)** The T-TDSM can be filtered considering several groups of amino acids to evaluate their role for a certain application (Section 2.2.). (**E)** The non-stochastic tensor is raised to the kth power (−12 to 12) applying a Haddamard matrix product, to evaluate the interactions between amino acids (Section 2.4.). (**F**,**G)** The non-stochastic tensor can be normalized using the simple stochastic and the mutual probability methods, respectively. (Section 2.5.). (**H)** The total tensor can be split into amino acid-based tensors (Section 2.1.). (**I**) The application of N-algebraic forms allows the transformation of the extracted information present on the macromolecular vectors and the tensors (Section 2.1.). (**J)** The obtained amino acid-based indices are stored in a Local Amino Acidic Invariant (LAI) (Section 2.1.). (**K)** The use of aggregation operators is proposed as a fusion operation for the LAI (Section 2.3.)

Consequently, if a protein contains “B” *aa* in its structure, the T-TDSM (${{\mathbb{Z}}}^{k}$) can be expressed as the sum of “B” *aa*-level matrices (${{\mathbb{Z}}}^{aa,k}$) (see Fig. 2). From this concept, after the application of algebraic maps on every A-TDSM, we will obtain “B” *aa*-level indices, denoted as ${}_{tr}L_{aa}$ (see Eq. (3)), which will be stored on an array (see Fig. 3).

This array will be designated as LAI (Local Amino Acidic Invariant) as a correspondence of the LOVI vector for organic molecules (Local Vertex Invariant)^60,61^. From the LAI vector, the total (whole-protein) three-linear indices can be calculated by using aggregation operators (which is a generalization concept for merging components)^62^. These aggregation operators will be discussed in Section 2.3. The general calculation scheme for these novel biomacro-molecular indices is shown in Fig. 3.

### Definition for the group-based 3D protein MDs considering three-linear forms

If we consider clusters of *aa* classified in terms of their activity/properties on solution or their probability to generate a certain secondary structure (see Table 1), group-based indices can be computed by choosing the selected *aa*-based indices stored in the LAI. Consequently, a new vector denominated Local Group-based Amino Acidic Invariant (LAI_G_) is generated. Considering the concept of aggregator operators, a new type of general indices based on *aa* groups could be generated. This operation allows to evaluate the influence of certain *aa* in a variety of applications on protein science.

**Table 1:** Amino acids groups considered for the computation of the novel 3D algebraic biomacro-molecular descriptors for proteins.

Group	Amino acids
FAH^a^	ALA, CYS, LEU, MET, GLU, GLN, HIS, LYS.
FBS^b^	VAL, ILE, PHE, TYR, TRP, THR.
UFG^c^	GLY, PRO.
AFT^d^	GLY, SER, ASP, ASN, PRO.
ALG^e^	GLY, ALA, PRO, VAL, LEU, ILE, MET.
ARO^f^	PHE, TYR, TRP.
RPC^g^	LYS, HIS, ARG.
RNC^h^	ASP, GLU.
RAP^i^	PRO, ILE, ALA, VAL, LEU, PHE, TRP, MET.
RPU^j^	ASN, CYS, GLY, SER, THR, TYR, GLN.

^a^Alpha helix favoring amino acids; ^b^Beta-sheets favoring amino acids; ^c^Unfolding amino acids; ^d^Beta-turn favoring amino acids; ^e^Aliphatic; ^f^Aromatic; ^g^Polar positively charged; ^h^Polar negatively charged; ^e^Apolar; ^j^Polar uncharged.

### Generation of novel protein mds from amino acid-based indices using aggregation operators

An invariant could be defined as a generalization procedure for merging different components to obtain one fused expression. The hypothesis that the most appropriate global definition of a natural system may not necessarily be additive is our initiative to propose this tool as an alternative for the generation of MDs. As proof of the concept, in the work done by Barigye *et al*.^62^, it was demonstrated that other operators besides the sum could yield better correlations with determined chemical properties. These invariants (aggregator operators) are classified in four major groups that are presented as follows: (i) **Norms (or Metrics) Invariants:** Minkowski norms (N1, N2, N3). *Note that the N1 corresponds to the linear combination (summation) of the elements in LAI*; (ii) **Mean Invariants (first statistical moments):** Geometric mean (G), arithmetic mean (M), quadratic mean (P2), power mean of third degree (P3) and harmonic mean (A); (iii) **Statistical Invariants (highest statistical moments):** Variance (V), skewness (S), kurtosis (K), standard deviation (SD), variation coefficient (CV), range (R), percentile 25 (Q1), percentile 50 (Q2), percentile 75 (Q3), inter-quartile range (I50), maximum _tr_L (MX) and minimum _tr_L (MN); and iv) **Classical Invariants:** Autocorrelation (AC), Gravitational (GV), Total Information Content (TIC), Mean Information Content (MIC), Standardized Information Content (SIC), Total Sum (TS) and Kier-Hall Connectivity (KH).

These invariants are applied to the LAI vector that contains the *aa* based indices as a strategy to obtain a series of global (or local: aa-based or group-based) indices that could contain orthogonal information from the use of the metric invariant N1. A Table indicating all formulae for the aggregation operators proposed is indicated on SMI-B.

### Definition of the three-tuple-(Dis) similarity matrix (TDSM) for physicochemical information extraction

Macro-molecular graphs allow the study of chemical interactions in biological systems to obtain more information on the behavior shown on experimental observations^63,64^; protein geometric (3D) representations indicate the distribution of its constituent amino acids in space. It is important to mention that the stability and maintenance of this complex structure relies on the inter-residue interactions^65^. Regarding this graphical approach, the *aa* on the protein can be considered as pseudo-vertices, which possess spatial coordinates defined by a chosen carbon representation. Alpha carbon (C_α_) has been the most used representation for protein geometrical/topological studies^12,15,64,66^, however, there were studies where Beta Carbon (C_β_) was considered as a simple atom(pseudo-node)-based representation^67^.

In this report, we propose two additional representations (Amide Carbon (AB) and the average of the coordinates of all atoms in the amino acid (AVG)) to observe the behavior and information content that these representations could bring respect the other existing representations. Furthermore, all interactions and bonding between these pseudo vertices are considered as connections between them. Here, all these interactions between amino acids will be computed by considering relationships (multi-metrics) among three *aa* $({z}_{ijl}^{k})$. Therefore, three-tuple spatial-(dis)similarity matrices $({{\mathbb{Z}}}^{k})$ will be generated as a representation of the bio-macro-molecular structure.

**Figure 4:**
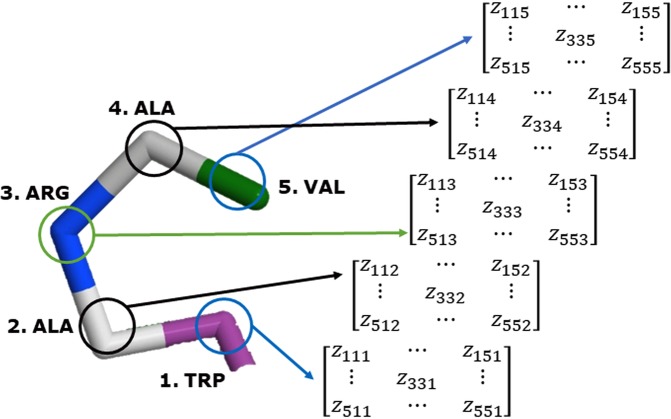
Computation of the Three-Tuple-(Dis) Similarity Matrix (TDSM) for an example truncated peptide (5WRX). Z_ijl_ is the value resulting of the use of a multi-metric (Bond Angle, Triangle Perimeter) (see Table 2). The obtained tensor has n × n × n dimensions, where n is the number of amino acids on the protein.

The formal definitions of elements ${z}_{ijl}^{k}$ of the matrix ${{\mathbb{Z}}}^{k}$ are indicated as follows (see Eq. (5)) (See Fig. 4):

5 $$\begin{array}{rcl}{z}_{ijl}^{k} & = & \begin{array}{cc}T{T}_{ijl} & {\rm{if}}\,{\bf{i}}\wedge {\bf{j}}\wedge {\bf{l}}\,are\,not\,equal\end{array}\\  & = & \begin{array}{cc}{D}_{ijl} & {\rm{if}}\,{\bf{i}},{\bf{j}}\vee {\bf{j}},{\bf{l}}\vee {\bf{i}},{\bf{l}}\,are\,equal\end{array}\\  & = & \begin{array}{cc}0 & {\rm{otherwise}}\end{array}\end{array}$$

where, $T{T}_{ijl}$ is a measure for ternary relations of amino acids (multi-metric), ${D}_{ijl}$ is a measure for duplex relation of amino acids (metric between 2 amino acids).

**Figure 5:**
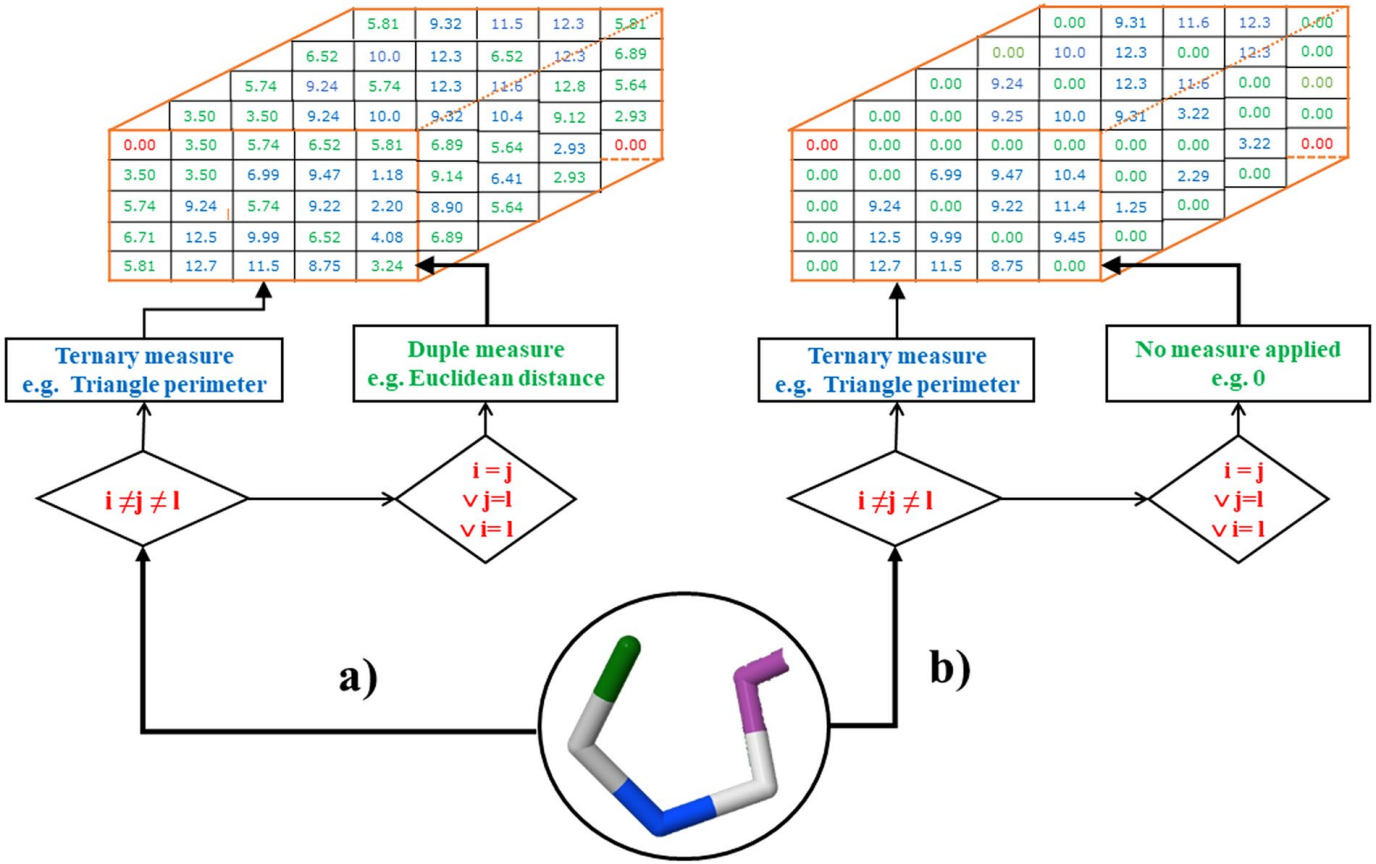
Selection of multi-metrics or metrics for the definition of the Three-Tuple-(Dis) Similarity Matrix (TDSM) on the truncate peptide 5WRX by using AB representation. A multi-metric is considered (**a**) Complete when it considers not only the relationships between 3 amino acids (multi-metrics, here *Triangle Perimeter*), but also relationships between 2 amino acids (metrics, here *Euclidean Distance*). A multi-metric is considered (**b**) Non-Complete when it considers only the relationships between 3 amino acids (relationships between 2 amino acids are defined as zero in the TDSM). Moreover, the diagonal of the tensor (conformed by all the tensor elements where i = j = l), could have zero values if the measure was applied considering every *aa* as a reference or they could be different from zero values if the measure was applied considering the center of mass of the protein.

From Eq. (5) we can observe that, when the *aa i*, *j* or *l* on the protein are different, the measure used for calculation is ternary. The ternary measures used for the computation of the indices are indicated in Table 2. However, when a multi-metric cannot be computed (two *aa* are the same), then it could be reduced to an inferior measure (duplex relation). The duplex measures used for the computation are indicated in SMI-C. It is important to remark that when a ternary measure is selected to codify the information of the protein, is mandatory to select at least one duplex measure or metric. Nevertheless, the selection of a metric is not mandatory when the ternary measures are related to the Volume, Bond Angle and Dihedral Angle measures (see Fig. 5).

**Table 2:** Multi-metrics available for the calculation of the novel 3D algebraic MDs for proteins. In bold, the software ID number of the multi-metric is indicated.

Measure	Formula	Symmetry
**Ternary Measures** ***(T***_***XYZ***_ ***)*** **Geometric-based**		
Triangle Area (**M33-M34**)	$$\begin{array}{rcl}{T}_{XYZ} & = & \sqrt{s(s-{d}_{XY})(s-{d}_{YZ})(s-{d}_{ZX})}\\ s & = & \frac{{d}_{XY}+{d}_{YZ}+{d}_{ZX}}{2}\end{array}$$	S
Triangle’s Incircle Area (**M35-M36**)	${T}_{XYZ}=\pi {(\frac{2\sqrt{s(s-{d}_{XY})(s-{d}_{YZ})(s-{d}_{ZX})}}{{d}_{XY}+{d}_{YZ}+{d}_{ZX}})}^{2}$	S
Summation Sides (**M37-M38**)	*T*_*XYZ*_ = *d*_*XY*_ + *d*_*YZ*_	A
Bond angle (Angle between sides) (**M39-M40**)	${A}_{X},{A}_{Y},{A}_{Z}\,coordinates\,of\,three\,aminoacids\,of\,a\,protein$ $$\begin{array}{rcl}U & = & {A}_{X}-{A}_{Y},V={A}_{Z}-{A}_{Y}\\ {T}_{XYZ} & = & \alpha =\arccos (\frac{U\ast V}{|U|\ast |V|})\end{array}$$	A
**Ternary Measures** ***(T***_***XYZ***_ ***)*** **(Cluster-Similarity-based)**		
MIN-RULE [1-Nearest neighbor (NN)] (**M41-M42**)	${T}_{1XYZ}=\,{\min }({d}_{XZ},{d}_{YZ})$ $${V}_{2}=\{\begin{array}{c}Y,{d}_{XY} < {d}_{XZ}\\ Z,otherwise\end{array}$$ $$\,{V}_{3}({V}_{2})=\{\begin{array}{l}Y,Y\ne {V}_{2}\\ Z,otherwise\end{array}$$ ${T}_{2XYZ}=\,{\rm{\min }}({d}_{X{V}_{3}},{d}_{{V}_{2}{V}_{3}})$	A
JOIN-RULE (2-NN) **(M43-M44)**	$d\,{\min }\,{({d}_{XY},{d}_{YZ},{d}_{ZX})}_{min}$ $d\,{\max }\,{({d}_{XY},{d}_{YZ},{d}_{ZX})}_{max}$ $$join({d}_{XY},{d}_{YZ},{d}_{ZX},{d}_{{\rm{\min }}},{d}_{{\rm{\max }}})=\{\begin{array}{c}{d}_{XY},{d}_{{\rm{\min }}} > {d}_{XY} < {d}_{{\rm{\max }}}\\ {d}_{YZ},{d}_{{\rm{\min }}} > {d}_{YZ} < {d}_{{\rm{\max }}}\\ {d}_{ZX},{d}_{{\rm{\min }}} > {d}_{ZX} < {d}_{{\rm{\max }}}\end{array}$$ ${T}_{XYZ}=join({d}_{XY},{d}_{YZ},{d}_{ZX},dma{x}_{min})$	S
MAX-RULE (Furthest neighbor) (**M45-M46**)	${T}_{XYZ}=\,{\max }({d}_{XZ},{d}_{YZ})$	A
AVE-RULE (Average-link) **(M47-M48)**	${T}_{XYZ}=\frac{{d}_{XZ}+{d}_{YZ}}{2}$	A
MED-RULE (**M49-M50**)	${T}_{XYZ}=\frac{{d}_{XZ}+{d}_{YZ}}{2}-\frac{{d}_{XY}}{4}$	A
WAR-RULE (**M51-M52**)	${T}_{XYZ}={d}_{X\bar{C}}^{2}+{d}_{Y\bar{C}}^{2}+{d}_{Z\bar{C}}^{2}-{d}_{X\bar{C}XY}^{2}-{d}_{Y\bar{C}XY}^{2}$	A
ADJ-RULE (**M53-M54**)	${T}_{XYZ}=\,{\max }({d}_{XY},{d}_{YZ},{d}_{ZX})-{d}_{XY}$	A
MAH-RULE Similarity with the Ward’s method (**M55-M56**)	${T}_{XYZ}={d}_{X\bar{C}}^{M\,2}+{d}_{Y\bar{C}}^{M\,2}+{d}_{Z\bar{C}}^{M\,2}-{d}_{X\bar{C}XY}^{M\,2}-{d}_{Y\bar{C}XY}^{M\,2}$	
**Ternary Measures** ***(T***_***XYZ***_***)*** **Classic-**, **Data-fusion- and Statistics- (Operators-based)**		
ADD-RULE (Average D/D degree) (**M57**-**M58**)	${T}_{XYZ}=\frac{1}{3}(\frac{{d}_{XY}}{{p}_{XY}}+\frac{{d}_{YZ}}{{p}_{YZ}}+\frac{{d}_{ZX}}{{p}_{ZX}})$	S
SUM-RULE (Wiener index) (**M59-M60**)	${T}_{XYZ}={d}_{XY}+{d}_{YZ}+{d}_{ZX}$	S
PRO-RULE (**M61-M62**)	${T}_{XYZ}={d}_{XY}\cdot {d}_{YZ}\cdot {d}_{ZX}$	S
QUA-RULE (**M63-M64**)	${T}_{XYZ}={(\frac{{{d}_{XY}}^{2}+\cdot {{d}_{YZ}}^{2}+{{d}_{ZX}}^{2}}{3})}^{\frac{1}{2}}$	S
GEO-RULE (**M65-M66**)	${T}_{XYZ}={(\frac{{{d}_{XY}}^{3}+\cdot {{d}_{YZ}}^{3}+{{d}_{ZX}}^{3}}{3})}^{\frac{1}{3}}$	S
RAN-RULE (**M67-M68**)	${T}_{XYZ}=\,{\max }({d}_{XY},{d}_{YZ},{d}_{ZX})-\,{\min }({d}_{XY},{d}_{YZ},{d}_{ZX})$	S
**Ternary Measures** ***(T***_***XYZ***_ ***)*** **Agreement Coefficients-based**		
IC-RULE Additivity-Corrected (**M69-M70**)	${T}_{XYZ}=\frac{2({S}_{XY}+{S}_{XZ}+{S}_{YZ})}{2({{S}_{X}}^{2}+{{S}_{Y}}^{2}+{{S}_{Z}}^{2})+{(\bar{X}-\bar{Y})}^{2}+{(\bar{X}-\bar{Z})}^{2}+{(\bar{Y}-\bar{Z})}^{2}}$	A
AC-RULE Aditividad-corregida (**M71-M72**)	${T}_{XYZ}=\frac{{S}_{XY}+{S}_{XZ}+{S}_{YZ}}{{{S}_{X}}^{2}+{{S}_{Y}}^{2}+{{S}_{Z}}^{2}}$	S
PC-RULE Proportionality-Corrected (**M73-M74**)	${T}_{XYZ}=\sum _{i < j}^{k}\frac{({\sum }_{t}^{n}{U}_{it}{U}_{jt}-n\overline{{U}_{i}}\overline{{U}_{t}})/A}{\frac{k}{2}(k-1)-n{\sum }_{i < j}^{k}[\frac{\overline{{U}_{i}}\overline{{U}_{j}}}{A}]}$ $A={(\sum _{t}^{n}{{U}_{it}}^{2}\sum _{t}^{n}{{U}_{jt}}^{2})}^{\frac{1}{2}}$	S
LC-RULE Linearity-Corrected (mean pair-wise pearson correlation) (**M75-M76**)	${T}_{XYZ}=\frac{{r}_{XY}+{r}_{YZ}+{r}_{ZX}}{3}$	S

x ${\bar{C}}_{XYZ}({\bar{C}}_{XY})$ are the mean centroids for the atoms X, Y, Z (XY) in the protein, respectively, *d*^*M*^ is the Mahalanobis distance, *n* is the dimension (3), *k* is the number of combinations (i, j), when i < j [(1, 2) (1, 3) and (2, 3)], $\bar{U}$ is the arithmetic mean of the the variable *U*. The values of the subscript “***i***” (1, 2, 3) stands for the atoms (X, Y, Z), respectively (e.g for the combination (1, 2) U_1_ and U_2_ represent the atoms X and Y) and *r*_*XY*_ is the Pearson correlation between variables X and Y, p_XY_ is the topological distance between the amino acids containing atoms (X and Y).

There are two possibilities regarding the application of multi-metrics or metrics on the protein structure, these could be amino acid-based, or protein mass center-based. In the first option, the multi-metric is calculated considering the distance functions against every *aa*, consequently, the elements *z*_*ijl*_ of the T-TDSM when *i* = *j* = *l*, are zero. For the second case, the multi-metric is calculated considering the selected metric of each amino acid to the mass center of the protein, and all elements *z*_*ijl*_ on the T-TDSM are different from zero; this approach may offer a better discrimination among protein spatial structures given that it provides information about the centrality of *aa* residues.

**Figure 6:**
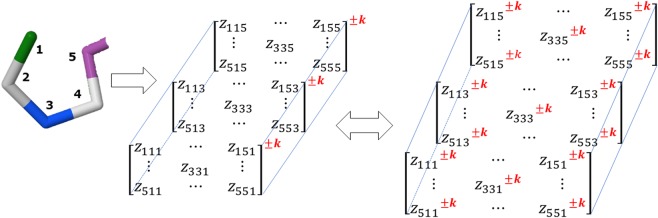
Application of the Hadamard Matrix Product on the Three-Tuple-(Dis) Similarity Matrix (TDSM) for the example truncated peptide 5WRX.

The *k*^*th*^ *three-tuple-(dis)similarity matrix* is obtained by performing a Hadamard matrix product^12^. This procedure performs the power operation in every element of the *three-tuple-(dis)similarity matrices*. The exponent *k* is a real number whose values can be positive or negative; when the parameter *k* is negative, the reciprocal operation is computed. This operation aims for the information extraction accounted by the intra-molecular forces that occur in the protein structure due the residues present in every *aa*. The range of values to evaluate this product could be from −12 to 12, e.g. *k* = −1 is related to the gravitational potential, *k* = −2 is related to the Coulomb potential (See Fig. 6 for more details).

When normalizing procedures are not employed (see below section 2.6) for the elements of ${{\mathbb{Z}}}^{k}$, these matrices are designed as the *k*^*th*^ *non-stochastic three-tuple-(dis)similarity matrices (NS-T-TDSM)* $({}_{ns}{\mathbb{Z}}_{k})$.

### Probabilistic transformations of the TDSM

Although normalization methods for geometrical matrices are not usually employed, there are several descriptors which use this concept for organic molecules and RNA secondary structures, protein sequences and viral surfaces^68–72^. There are advantages of using normalized matrices such as information standardization and as a tool for the computation of different *k*^*th*^ three-linear MDs^25^.

Since probabilistic transformations have only been applied for two-tuple matrices, a generalization for these concepts will be used to normalize the *k*^*th*^ *non-stochastic three-tuple-(dis)similarity matrices* obtained from the computation described computation above. In this study, two probability schemes could be applied: a) simple stochastic and b) mutual probability transformations.

**Figure 7:**
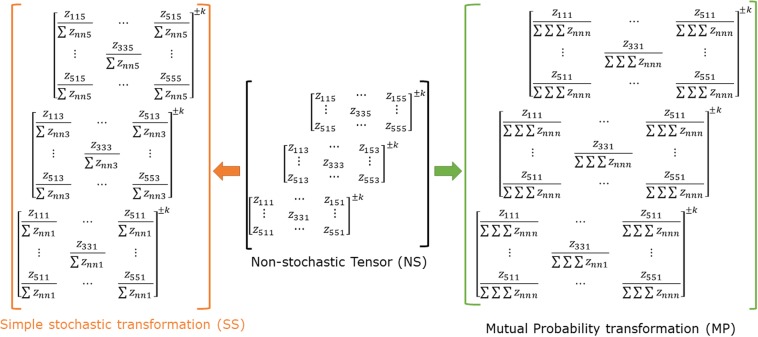
Application of probabilistic transformations on the Three-Tuple-(Dis) Similarity Matrix (TDSM). The simple stochastic transformation (SS) consists on dividing every element of a 2D matrix for the sum of all elements in that 2D matrix. The mutual probability procedure consists on dividing every element of a 2D matrix for the sum of all elements in the tensor (3D matrix).

The *k*^*th*^ *simple-stochastic three-tuple-(dis)similarity matrices* ${}_{ss}{\mathbb{Z}}_{k}$ (SS-T-TDSM) and *k*^*th*^ mutual probability *three-tuple-(dis)similarity matrices* ${}_{mp}{\mathbb{Z}}_{k}$ (MP-T-TDSM), which are obtained from ${}_{ns}{\mathbb{Z}}_{k}$, have been defined as follows:

6 $${}_{ss}z_{i\,jl}^{k}=\frac{{}_{ns}z_{i\,jl}^{k}}{{S}_{jl}}=\frac{{}_{ns}z_{i\,jl}^{k}}{{\sum }_{j=1}^{n}\,{\sum }_{k=1}^{n}\,{}_{ns}z_{i\,jl}^{k}}$$

7 $${}_{mp}z_{ijl}^{k}=\,\frac{{}_{ns}z_{ijl}^{k}}{{S}_{ijl}}=\frac{{}_{ns}z_{ijl}^{k}}{{\sum }_{i=1}^{n}\,{\sum }_{j=1}^{n}\,{\sum }_{k=1}^{n}\,{}_{ns}z_{ijl}^{k}}$$

where, ${}_{ns}z_{ijl}^{k}$ are the elements of the *k*^th^ *non-stochastic three-tuple-(dis)similarity matrices*. S_jl_ is the summation of all entries of the two-tuple matrix corresponding to each *aa i* in a three-tuple matrix for the simple stochastic case whereas for the mutual probability scheme, S_ijl_ is the summation of all elements of the tensor ${}_{ns}{\mathbb{Z}}_{k}$ (see Fig. 7).

### Computational calculation of the new proposed protein MDs

These novel 3D algebraic MDs can be generated by using the *in-house* software MuLiMs MCoMPAs (at ToMoCoMD-CAMPS system), an open access java-based software. The software allows the user to evaluate all the theoretical configurations presented above and it is available at http://www.tomocomd.com/; it runs on all operative systems available and it presents two versions, a graphical user interface (GUI) version and console version for calculations on a high-performance computing system (HPC).

## Application Of The N-Linear 3d Algebraic Biomacro-Molecular Descriptors To The Prediction Of Folding Rate And Scop Structural Classification Of Proteins

### Benchmark datasets

The training set used for the modelling of the folding rate of proteins (80 proteins) was proposed by Ouyang^31^. It is important to mention that the case “2BLM” was removed from the set since this case considers only the alpha carbon representation. The test set used here (17 proteins) was proposed by Ruiz-Blanco^36^.

The set used for protein structural classification (204 proteins) was proposed by K.C. Chou based on the SCOP classification (52 all alpha, 61 all beta, 45 alpha/beta and 46 alpha + beta)^39^. This set was divided into two groups, 149 proteins were used for the training set and 55 were used for the test set. The details about how this separation was done could be found in Marrero-Ponce *et al*.^40^ (see also section **3.1)**. The structures (pdb files) of the protein and the respective protein representations (pdbx files) could be found as SMII-1 and SMII-2.

### Novel 3D algebraic MDs calculation and dimensionality reduction

The software **MuLiMs-MCoMPAs** (acronym for Multi-Linear Maps based on N-Metric & Contact Matrices of 3D-Protein and Amino-Acids Weighting**s**) belonging to the ToMoCoMD-CAMPS suite (acronym for *TOpological MOlecular COMputational Design-Computed-Aided Modelling in Protein Science*) allows the computation of these novel protein descriptors. However, in order to reduce the number of MDs to evaluate, analysis of collinearity between indices and information redundancy were performed to obtain 10 suggested theoretical configurations (here designed as *projects*). The projects designed and used in the present study are shown in SMII-3. From these projects, a total of 20.263 MDs were generated on an HPC with the following computational characteristics: 16 cores Intel (R) Xeon (R) E5-2630 v3, 2.4 GHz of speed and 64 GB RAM using MuLiMs console version.

After the computation of the indices, additional dimensionality reduction procedures were performed. First, non-supervised and supervised procedures considering an information theoretic approach were employed for the reduction of the number of descriptors^73,74^. The software used for this purpose is known as IMMAN^75^. In addition to these reductions, a final supervised reduction was performed using subset filters which considered 2 search methods, Best First and Greedy Stepwise. The software used for this purpose was WEKA (version 3.8)^76^.

### Development of the regression and classification models

The folding rate modelling was performed using the software MOBYDIGS^77^, that combines Multiple Linear Regression (MLR) with a wrapper method based on Genetic Algorithm (GA). The GA was set up with the following considerations: population size: 100; reproduction/mutation rate show starts on 0.5 but it is changed from 0 to 1 while doing the exploration; selection method started on 0.5, but it was changed to 1 and 0 to evaluate more selection options. Several experiments were performed for the construction of models that considered only trilinear indices and the combination between trilinear and bilinear indices.

From the chosen test set, based on the prediction error obtained for all models, four proteins were excluded from the test set (outliers). These outliers were: pdb1jo8, pdb1spr_A, pdb1t8j, pdb2vik.

The protein structural classification was performed by using the software WEKA^76^, that combines the Linear Discriminant Analysis (LDA) with a subset method that uses two searching strategies: Best First and Greedy Stepwise, as well as a wrapper method. Several experiments were carried out for the generation of mathematical models that considered only trilinear indices and the combination between trilinear and bilinear indices.

#### Assessment of the models

Depending on the modelling technique, several statistical parameters were selected for the resulting mathematical expressions validation. Regarding the case of MLR, the leave one out cross validation (Q^2^_loo_) was used as a fitness function. The models were assessed as well considering the Y-scrambling (a(Q^2^))^78^ validation method and the bootstrapping technique (Q^2^_boot_)^79^, to reduce the possibility of casual correlation between the selected MDs and for the assessment of the predictive power of the models.

### Results and comparison with other approaches

The use of these novel biomacro-molecular descriptors for proteins as a main component for the generation of predictive mathematical models was proposed to evaluate the performance of these models against mathematical expressions generated using other MDs proposed in the literature. As a result, several models for the prediction of folding rate of proteins considering MLR as a modelling strategy and several models for the structural classification of proteins considering the SCOP dataset, using LDA as a modelling strategy, were obtained. The best ranked models and the comparison table are shown below.

#### Folding rate evaluation

This section presents the equations and statistical parameters for the best two models obtained for folding rate prediction considering only trilinear indices (Eqs 8 and 9) and the best two models obtained for folding rate prediction considering the combination of the trilinear and bilinear indices (Eqs 10 and 11). These equations are presented below:

8 $$\mathrm{ln}\,({\rm{k}})=0.0123\ast {\rm{A}}-0.0315\ast {\rm{B}}+{\rm{19.100}}\ast {\rm{C}}+7029.6\ast {\rm{D}}$$

where,

A = AVG_TS[7]_N1_Tr_M33(M3)_MP-8_o_RPU_KA_PAH-ISA-HWS_MCoMPAs

B = AVG_N3_TrQB_M55(M15)_SS-2_T_KA_PAH-ISA_MCoMPAs

C = AVG_Q1_TrC_M58(M15)_SS0_T_KA_PAH_MCoMPAs

D = AVG_GV[5]_MX_TrF_M41(M5)_MP7_o_T_KA_PBS_MCoMPAs

9 $$\mathrm{ln}\,({\rm{k}})=-0.0323\ast {\rm{A}}+20.3011\ast {\rm{B}}+7205.01\ast {\rm{C}}-1.7572$$

where,

A = AVG_N3_TrQB_M55(M15) _SS-2_T_KA_PAH-ISA_MCoMPAs

B = AVG_Q1_TrC_M58(M15) _SS0_T_KA_PAH_MCoMPAs

C = AVG_GV[5]_MX_TrF_M41(M5) _MP7_o_T_KA_PBS_MCoMPAs

10 $$\mathrm{ln}\,({\rm{k}})=-44766.6\ast {\rm{A}}-0.96157\ast {\rm{B}}+0.20729\ast {\rm{C}}-3.25903\ast {\rm{D}}+25.4265$$

where,

A = CB_Q2_B_M19_NS-3_T_LGP[ + 12.0]_LGL[4–11]_PAH-PBS_MCoMPAs

B = CB_K_Q_M5_NS-1_T_LGP[1-3]_KDS_MCoMPAs

C = CB_K_B_M2_SS-1_FBS_KA_MM-ECI_MCoMPAs

D = CB_MIC_N1_TrQB_M45(M8)_SS2_o_T_KA_PAH-Z3_MCoMPAs

11 $$\mathrm{ln}\,({\rm{k}})=-42920.3\ast {\rm{A}}+0.17709\ast {\rm{B}}-3.22386\ast {\rm{C}}+26.0880$$

where,

A = CB_Q2_B_M19_NS-3_T_LGP[ + 12.0]_LGL[4-11]_PAH-PBS_MCoMPAs

B = CB_K_B_M2_SS-1_FBS_KA_MM-ECI_MCoMPAs

C = CB_MIC_N1_TrQB_M41(M5)_SS2_o_T_KA_PAH-Z3_MCoMPAs

As can be observed from Table 3, the bootstrapping correlation coefficient Q^2^_boot_ calculated for each model presents a value greater than 0.73, which indicates the robustness of the calibrated models against perturbations over the training set. Moreover, the best ranked model was obtained with the combination of trilinear and bilinear indices and its Q2 value is 0.797 (Eq. 11). In addition, the parameters derived from Y-scrambling tests [a(Q^2^)] have in all cases values around −0.137, indicating low propensity to random correlations in predictions. Folding rate depends on the tridimensional structure and specific contact sites along the structure. The correlation obtained between the studied property and the set of proteins indicates that there is an increased amount of information related to the proposed descriptors. Consequently, it could be observed that these proposed descriptors extract orthogonal and novel information complementary to the bilinear algebraic indices. Regarding the composition of the indices that conform the equations, it can be observed that the protein representations C_β_ and AVG are present in all these models, indicating that these novel representations proposed extract more information that the Cα representation.

**Table 3:** Best models obtained for the folding rate prediction of 96 proteins using these novel molecular descriptors.

Model	Q^2^_LOO_	Q^2^_BOOT_	SDEP	Q^2^_EXT_ (w/outliers)	SDEPext (w/outliers)	Q^2^_EXT_ (w/o outliers)	SDEPext (w/o outliers)
***Trilinear indices-based models***							
**8**	77.79	76.57	2.035	34.16	3.180	82.37	2.938
**9**	74.80	73.83	2.167	32.28	3.170	85.75	2.786
***Bilinear and trilinear indices-based models***							
**10**	77.69	77.62	2.0392	60.87	2.387	79.57	2.964
**11**	79.70	79.26	1.9454	55.57	2.556	78.19	2.606

Furthermore, the similarity between the standard deviation (SDEP) values in training and test sets suggest that the obtained modes have a general applicability.

Regarding the statistical parameters obtained considering the external set of proteins (test set), the overall Q^2^_ext_ is higher than 0.78 (explains more than the 78% of the total variance), which indicates the high predictive capability of the models respect to this property. Moreover, the model with the highest Q^2^_ext_ is Eq. 10 with 0.86; this model was generated considering only trilinear indices. Based on the configuration of the descriptors used for the modelling, it could be observed that the mathematical tools such as operation aggregators (all the selected operators are different from the linear combination, which validates this theoretical statement), the normalization procedures (Simple stochastic and Mutual probability), steric physicochemical properties (PAH and PBS), and considering a protein mass center-based multi-metric and metric distance function calculation (which is a generalization that considers the whole protein structure), allowed a strong correlation between the indices and the response variable.

Concerning other MDs obtained to correlate the folding rate of proteins, it can be observed that the cross-validation correlation coefficient is the highest reported value for this application. Table 4 indicates all the values obtained for the training and test sets using the aforementioned descriptors. The values obtained in this study are superior to the value reported in the other reports.

**Table 4:** Comparison of the training and test set’s folding rate statistical parameters of several existing molecular descriptors for proteins against this approach.

Descriptors/Models	Descriptor Dimension	Cutoff Length	Q^2^ (%) (*training*)	SDEP (*training*)	Q^2^ (%) (*test*)	SDEP *(test)*
***From literature***						
Folding degree^36^	3D	—	73.96	2.20	54.76	2.03
Long Range Order^41^	3D	4	72.25	2.28	—	—
Contact order^15^	3D	2	73.96	2.19	—	—
Total Contact Distance^42^	3D	2	73.96	2.21	—	—
FoldRate web server^34^	1D	*	77.44	2.03	—	—
***This study***						
**Model 11**	3D	—	79.70	1.95	78.19	2.60
**Model 9**	3D	—	74.80	2.17	87.52	2.06

*Model constructed with an ensemble of mathematical equations.

Finally, all the best ranked models and its statistical parameters are indicated on SMIII-D.

#### Protein structural classification evaluation

The statistical values for the best four models obtained for SCOP protein structural classification are presented in Table 5; of which two of them are obtained with trilinear indices (Equations **12** and **13**), whereas the other two are obtained with combinations of trilinear and bilinear indices (Equations **14** and **15**).

**Table 5:** Best models obtained for the protein secondary structural classification of 204 proteins using these novel MDs.

Model	Representation	Number of Variables	Correct Classification (%) Training (149)	MCC Training	Correct Classification (%) Test (55)	MCC Test
***Trilinear indices-based models***						
**12**	Cβ	16	98.65	0.962	92.59	0.777
**13**	AVG, Cβ	19	95.97	0.884	89.09	0.718
***Bilinear and trilinear indices-based models***						
**14**	AB, Cβ, AVG	13	99.33	0.981	96.36	0.893
**15**	AB, Cβ, AVG	9	99.33	0.981	98.18	0.943

As it can be observed from Table 5, the overall number of variables in all the best models presented is between 9 and 19, suggesting that these training models have an high accuracy and a relatively low amount of variables on the prediction of structural classes regarding the training set. The best models obtained on the training set were equations (**14** and **15)** with an Acc. value of 99.33. It is important to mention that these models were obtained using the combination of trilinear and bilinear indices. Since the structural classification of proteins considers the amount of secondary structures (alpha helixes and beta sheets) present on the structure, the trilinear indices extract structural information in a higher degree than bilinear indices alone based on the results obtained. This statement can be supported by the generalizations applied on the mathematical definition of the indices, that allow more and non-redundant information from the protein structure.

Regarding the composition of the indices that conform the equations, it can be observed that the protein representations Cβ, AVG and AB are present in all these models, indicating that these novel representations proposed extract more information that the Cα representation.

Evaluating the MCC values for the training set, it can be observed that the values for all models are above 0.88, which indicates that the models have low classification errors due false positives and false negatives.

Regarding the results obtained for the external prediction, it can be observed that all models have a correct classification percentage above 89.09%, which indicates a high prediction value using the model resulting from the training set. The model with the highest prediction value is equation (**15)** with an Acc. value of 98.18%. The MCC value for this model is 0.943 which indicates a very low number of false positives and false negatives on the prediction.

Based on the configuration of the used descriptors on the classification models generated, it is possible to observe that several mathematical tools such as different metrics used for the definition of the distance between two amino acids, the local descriptors, and the use of several aggregation operators, allow better information extraction for this property classification models.

Concerning other descriptors generated to predict the secondary structural classification, the comparison between the reported statistical parameters used to evaluate the classification models using those descriptors and our models, it can be observed that the models proposed in this study have a higher classification percentage for the training and test sets (Table 6). All the best ranked models and its statistical parameters are indicated on SMIII-E.

**Table 6:** Comparison of the training set’s protein structural classification correct classification percentage of several existing molecular descriptors against this approach.

Descriptors/Models	Correct Classification (%) Training	Correct Classification (%) Test
***From literature***		
AA composition^13^	83.80	—
Pseudo AA composition^84^	91.20	—
Pair coupled AA composition^85^	74.50	—
PSI-BLAST^86^	94.10	—
Bilinear descriptors^40^	92.60	92.70
***This study***		
**Model 14**	99.33	96.36
**Model 15**	99.33	98.18

## Conclusion and Future Research

The definition of a new type of 3D MDs based on N-linear algebraic forms allowed the codification of geometrical and topological information regarding relationships between three amino acids on a protein by the evaluation and comparison of the selected statistical parameters obtained for two representative applications in protein science (folding rate and secondary structural classification). Consequently, these MDs constitute an alternative for the generation of proteins physicochemical properties’ and function predictive models.

Two new (AB and AVG) and two commonly used (C_α_ and C_β_) computing protein representations were evaluated for protein geometrical information extraction. Based on the results obtained from this study, it was observed that the higher information extraction was obtained when the proposed protein descriptors considered the beta carbon (C_β_) and the pseudo amino acid (AVG) representations.

As future research, we suggest using spherical truncating methods and generalized aggregation operators as another generalization strategy for the generation of these novel MDs. These mathematical tools could improve the information extraction from the proteins’ graphical representations.

Moreover, we suggest the evaluation of these novel biomacro-molecular descriptors for proteins in multi-reference studies (several representative protein science applications), that consider several benchmark data sets, to identify for what types of applications, these novel indices could perform better than the previous proposed approaches and how much orthogonal information can these molecular descriptors can obtain.

As pointed out in K.C. Chou’s review^80^ and demonstrated in a series of recent publications (see, e.g.^50,51,81^) user-friendly and publicly accessible web-servers represent the future direction for developing useful prediction methods and computational tools. Many webservers have significantly increased the impacts of bioinformatics on medical science^82^, driving medicinal chemistry into an unprecedented revolution^83^, we shall make efforts in our future work to provide a webserver for the topic presented in this paper.

## Supplementary information

SMI

SMIII

## Data Availability

The MuLiMs-MCoMPAs software and the respective user manual are freely available online at www.tomocomd.com.
