# Supplementary material for: Tensor Algebra-based Geometrical (3D) Biomacro-Molecular Descriptors for Protein Research: Theory, Applications and Comparison with other Methods
Source: Sci Rep. 2019 Aug 6;9:11391. doi: 10.1038/s41598-019-47858-2 (PMC6684663; doi:10.1038/s41598-019-47858-2)
Supplement: Supplementary file 1 — SMI [file 41598_2019_47858_MOESM1_ESM.docx]

**SUPPLEMENTARY MATERIAL I**

Tensor Algebra-based Geometrical (3D) Biomacro-Molecular Descriptors for Protein Research: Theory, Applications and Comparison with other Methods

**Julio E. Terán, Yovani Marrero-Ponce, (**
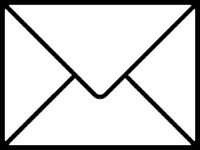
**) Ernesto Contreras-Torres, César R. García-Jacas, Ricardo Vivas-Reyes, Enrique Terán and F. Javier Torres**

***Corresponding author*** (
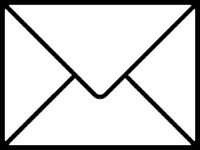
):

**Yovani Marrero-Ponce**

[ymarrero77@yahoo.es](mailto:ymarrero77@yahoo.es)

INDEX

SUPPLEMENTARY MATERIAL I (SMI)

[**SMI-A**. Physicochemical properties for the 20 elemental amino acids 3](#_Toc536512790)

[**SMI-A** Physicochemical properties for the 20 elemental amino acids (*Continued*) 4](#_Toc536512791)

[**SMI-B.** Aggregation operators Formulae 5](#_Toc536512792)

[**SMI-B.** Aggregation operators Formulae (*Continued*) 6](#_Toc536512793)

[**SMI-B.** Aggregation operators Formulae (*Continued*) 7](#_Toc536512796)

[**SMI-B.** Aggregation operators Formulae (*Continued*) 8](#_Toc536512805)

[**SMI-C.** Metrics used for duplex relations. 9](#_Toc536512812)

**SMI-A**. Physicochemical properties for the 20 elemental amino acids

| **Amino acid** | **Code** | **z-scale^a^** | | | **ISA^b^** | **ECI^c^** | **PIE^d^** | **HWS^e^** | **KDS^f^** |
| --- | --- | --- | --- | --- | --- | --- | --- | --- | --- |
|  |  | **z_1_** | **z_2_** | **z_3_** |  |  |  |  |  |
| Alanine | ALA | 0.01 | -1.73 | 0.09 | 62.9 | 0.05 | 6.01 | -0.5 | 1.8 |
| Arginine | ARG | 2.88 | 2.52 | -3.44 | 52.98 | 1.69 | 10.76 | 3 | -4.5 |
| Asparagine | ASN | 3.22 | 1.45 | 0.84 | 17.87 | 1.31 | 5.41 | 0.2 | -3.5 |
| Aspartate | ASP | 3.64 | 1.13 | 2.36 | 18.46 | 1.25 | 2.77 | 3 | -3.5 |
| Cysteine | CYS | 0.71 | -0.97 | 4.13 | 78.51 | 0.15 | 5.07 | -1 | 2.5 |
| Glutamate | GLU | 3.08 | 0.39 | -0.07 | 30.19 | 1.31 | 3.22 | 0.2 | -3.5 |
| Glutamine | GLN | 2.18 | 0.53 | -1.14 | 19.53 | 1.36 | 5.65 | 3 | -3.5 |
| Glycine | GLY | 2.23 | -5.36 | 0.3 | 19.93 | 0.02 | 5.97 | 0 | -0.4 |
| Histidine | HIS | 2.41 | 1.74 | 1.11 | 87.38 | 0.56 | 7.59 | -0.5 | -3.2 |
| Isoleucine | ILE | -4.44 | -1.68 | -1.03 | 149.77 | 0.09 | 6.02 | -1.8 | 4.5 |
| Leucine | LEU | -4.19 | -1.03 | -0.98 | 154.35 | 0.01 | 5.98 | -1.8 | 3.8 |
| Lysine | LYS | 2.84 | 1.41 | -3.14 | 102.78 | 0.53 | 9.74 | 3 | -3.9 |
| Methionine | MET | -2.49 | -0.27 | -0.41 | 132.22 | 0.34 | 5.74 | -1.3 | 1.9 |
| Phenylalanine | PHE | -4.92 | 1.3 | 0.45 | 189.42 | 0.14 | 5.48 | -2.5 | 2.8 |
| Proline | PRO | -1.22 | 0.88 | 2.23 | 122.35 | 0.16 | 6.48 | 0 | -1.6 |
| Serine | SER | 1.96 | -1.63 | 0.57 | 19.75 | 0.56 | 5.68 | 0.3 | -0.8 |
| Threonine | THR | 0.92 | -2.09 | -1.4 | 59.44 | 0.65 | 5.87 | -0.4 | -0.7 |
| Tryptophan | TRP | -4.75 | 3.65 | 0.85 | 179.16 | 1.08 | 5.89 | -3.4 | -0.9 |
| Tyrosine | TYR | -1.39 | 2.32 | 0.01 | 132.16 | 0.72 | 5.66 | -2.3 | -1.3 |
| Valine | VAL | -2.69 | -2.53 | -1.29 | 120.91 | 0.07 | 5.97 | -1.5 | 4.2 |
| ^a^Z-scale (Hellberg et al., 1987), ^b^Side-chain isotropic surface area (Collantes and Dunn III, 1995), ^c^Atomic charge (Collantes and Dunn III, 1995), ^d^Isoelectric point (Hellberg et al., 1987), ^e^Hoop-Woods hydropathy index (Hopp and Woods, 1981), ^f^Kyte-Doolittle hydropathy index (Kyte and Doolittle, 1982). | | | | | | | | | |

**SMI-A** Physicochemical properties for the 20 elemental amino acids (*Continued*)

| **Amino acid** | **Code** | **MV^g^** | **PAH^h^** | **PBS^i^** |
| --- | --- | --- | --- | --- |
| Alanine | ALA | 88.6 | 1.29 | 0.9 |
| Arginine | ARG | 173.4 | 0.96 | 0.99 |
| Asparagine | ASN | 114.1 | 0.9 | 0.76 |
| Aspartate | ASP | 111.1 | 1.04 | 0.72 |
| Cysteine | CYS | 108.5 | 1.11 | 0.74 |
| Glutamate | GLU | 143.8 | 1.44 | 0.75 |
| Glutamine | GLN | 138.4 | 1.27 | 0.8 |
| Glycine | GLY | 60.1 | 0.56 | 0.92 |
| Histidine | HIS | 153.2 | 1.22 | 1.08 |
| Isoleucine | ILE | 166.7 | 0.97 | 1.45 |
| Leucine | LEU | 166.7 | 1.3 | 1.02 |
| Lysine | LYS | 168.6 | 1.23 | 0.77 |
| Methionine | MET | 162.9 | 1.47 | 0.97 |
| Phenylalanine | PHE | 189.9 | 1.07 | 1.32 |
| Proline | PRO | 112.7 | 0.52 | 0.64 |
| Serine | SER | 89 | 0.82 | 0.95 |
| Threonine | THR | 116.1 | 0.82 | 1.21 |
| Tryptophan | TRP | 227.8 | 0.99 | 1.14 |
| Tyrosine | TYR | 193.6 | 0.72 | 1.25 |
| Valine | VAL | 140 | 0.91 | 1.49 |
| ^g^Side-chain amino acid volume (Zamyatnin, 1972), ^h,i^ Relative frequencies with which an amino acid appear forming α-helices, and β-sheets, respectively (Mathews et al., 2000) | | | | |

**SMI-B.** Aggregation operators Formulae

| **No.** | **Group** | **Name** | **ID** | **Formula** |
| --- | --- | --- | --- | --- |
| 1 | Norms (Metrics) | Minkowski norm (p = 1) | N1 | \| $\text{N1}=\sum_{a=1}^{n} L_{a}$ \| \| --- \| \| |
|  |  | Manhattan norm |  |  |
| 2 |  | Minkowski norm (p = 2) | N2 | \| $\text{N2}=\sqrt{\sum_{a=1}^{n} {L_{a}}^{2}}$ \| \| --- \| \| |
|  |  | Euclidean norm |  |  |
| 3 |  | Minkowski norm (p = 3) | N3 | \| $\text{N3}=\sqrt[3]{\sum_{a=1}^{n} {L_{a}}^{3}}$ \| \| --- \| |
| 4 |  | Penrose size | PN | $\text{PN}=\sqrt{\frac{1}{n^{2}}\left[ \sum_{a=1}^{n} (L_{a}) \right]^{2}}$ |

**SMI-B.** Aggregation operators Formulae (*Continued*)

| **No.** | **Group** | **Name** | **ID** | **Formula** |
| --- | --- | --- | --- | --- |
| 5 | Mean  (first  statistical moment) | Geometric Mean | GM | $G=\sqrt[n]{\prod_{a=1}^{n} L_{a}}$ |
| 6 |  | Arithmetic Mean | AM | $M_{\beta}=\left( \frac{L_{1}^{\beta}+L_{2}^{\beta}+...+L_{n}^{\beta}}{n} \right)^{\frac{1}{\beta}}$ |
|  |  | (Power mean of degree β = 1) |  |  |
| 7 |  | Quadratic Mean | P2 |  |
|  |  | (Power mean of degree β = 2) |  |  |
| 8 |  | Power mean of degree β = 3 | P3 |  |
| 9 |  | Harmonic Mean | A |  |
|  |  | (Power mean of degree β = -1) |  |  |

**SMI-B.** Aggregation operators Formulae (*Continued*)

| **No.** | **Group** | **Name** | **ID** | **Formula** |
| --- | --- | --- | --- | --- |
| 10 | Statistical  (highest statistical moments) | Skewness | S | $S=\frac{n*\left( X_{3} \right)}{(n-1)(n-2)(DE)^{3}}$  $X_{3}=\sum_{a=1}^{n} (L_{a}-AM)^{3}$   \|  \| \| --- \| \| |
| 11 |  | Variance | V | $V=\frac{\sum_{a=1}^{n} \left( L_{a}-M \right)^{2}}{n-1}$ |
| 12 |  | Kurtosis | $K=\frac{n\left( n+1 \right)X_{4}-3\left( X_{2} \right)\left( X_{2} \right)\left( n-1 \right)}{(n-1)(n-2)(n-3)(DE)^{4}}$  $X_{j}=\sum_{a=1}^{n} (L_{a}-AM)^{j}$   \| K \| \| --- \| \| |  |
| 13 |  | Standard Deviation | DE | $\text{DE}=\sqrt{\frac{\left( \sum L_{a}-AM \right)^{2}}{n-1}}$ |
| 14 |  | Variation Coefficient | CV | $\text{CV}=\frac{DE}{AM}$ |
| 15 |  | Range | R | $R={L\min}_{\max}$ |
| 16 |  | Percentile 25 | Q1 | $\text{Q1 }=\left[ \frac{N}{4}+\frac{1}{2} \right]$ |
| 17 |  | Percentile 50 | Q2 |  |
| 18 |  | Percentile 75 | Q3 | $\text{Q2 }=\left[ \frac{N}{2}+\frac{1}{2} \right]$  $\text{Q3 }=\left[ \frac{3N}{4}+\frac{1}{2} \right]$ |
| 19 |  | Inter-quartile Range | I50 | $\text{I50}=Q3-Q1$ |
| 20 |  | Maximum value | MX | MX = _tr_*L* max |
| 21 |  | Minimum value | MN | MN = _tr_*L* min |

**SMI-B.** Aggregation operators Formulae (*Continued*)

| **No.** | **Group** | **Name** | **ID**  $AC_{k}=\sum_{i=1}^{n} \sum_{j\geq1}^{n} L_{i}\times L_{j}\bullet(\delta(d_{ij},k))k=1,2,..7$ | **Formula** |
| --- | --- | --- | --- | --- |
| 22 | Classical | Autocorrelation | AC*^k^* |  |
| 23 |  | Gravitational | GV^k^ | $GV_{k}=\frac{1}{n}\sum_{i=1}^{n} \sum_{j=1}^{n} \frac{L_{i}L_{j}}{{{}^{k}d}_{ij}}\bullet\delta(d_{ij},k))k=1,2,..7$ |
| 24 |  | Total sum at lag k | TS*^k^* | $TS_{k}=\sum_{i=1}^{n} \sum_{j=1}^{n} L_{ij}\bullet\delta(d_{ij},k)) k=1,2,\ldots,7$ |
| 25 |  | Kier-Hall connectivity | KH*^m^* | ${}^{m}KH_{t}={\sum_{i=1}^{K} (\prod_{i=1}^{n_{k}} L_{i},w)}_{k}^{\lambda}$  *where, K is the number of sub-graphs, n_k_ is the number of amino acids in a group, λ is equal to ½, m and t are the sub-graph order and type, respectively* |
| 26 |  | Mean Information Content | MIC | $MIC=-\sum_{i=1}^{A} \frac{N_{g}}{N_{o}}\cdot\log_{2} \frac{N_{g}}{N_{o}}$  *where, Ng is the number of amino acids with the same LAI value. N_o_ is the number of amino acids in a molecule* |
| 27 |  | Total Information Content | TIC | $TIC=N_{0}\cdot\log_{2} N_{0}-\sum_{g=1}^{G} N_{g}\cdot\log_{2}N_{g}$ |
| 28 |  | Standardized Information Content | SIC | $SIC=\frac{IT}{N_{0}\cdot\log_{2} N_{0}}$ |

**SMI-C.** Metrics used for duplex relations.

| **Name** | **Formula** | **Range** |
| --- | --- | --- |
| Manhattan/ City-Block | $d_{\mathrm{XY}}=\sum_{j=1}^{n} \left\vert x_{j}-y_{j} \right\vert$ | $\left[ 0,\left. \infty\right) \right.$ |
| Euclidean | $d_{\mathrm{XY}}=\sqrt{\sum_{j=1}^{n} \left\vert x_{j}-y_{j} \right\vert^{2}}$ | $\left[ 0,\left. \infty\right) \right.$ |
| Chebyshev/Lagrange | $d_{\mathrm{XY}}=\max\left\{ \left\vert x_{j}-y_{j} \right\vert\right\}$ | $\left[ 0,\left. \infty\right) \right.$ |
| Bhattacharyya | $d_{\mathrm{XY}}=\sqrt{\sum_{j=1}^{n} \left( \sqrt{x_{j}}-\sqrt{y_{j}} \right)^{2}}$ | $\left[ 0,\left. \infty\right) \right.$ |
| Mahalanobis | $d_{\mathrm{XY}}=\sqrt{\left( X-Y \right)^{t}S^{-1}\left( X-Y \right)}$ | $\left[ 0,\left. \infty\right) \right.$ |
| Correlation | $d_{\mathrm{XY}}=1-\frac{\sum_{j=1}^{n} \left( x_{j}-\bar{X} \right)\left( y_{j}-\bar{Y} \right)}{\sqrt{\sum_{j=1}^{n} \left( x_{j}-\bar{X} \right)\sum_{j=1}^{n} \left( y_{j}-\bar{Y} \right)}}$ | $\left[ 0,2 \right]$ |
| Angular Separation | $d_{\mathrm{XY}}=1-\frac{\sum_{j=1}^{n} x_{j}y_{j}}{\sqrt{\sum_{j=1}^{n} x_{j}^{2}\sum_{j=1}^{n} y_{j}^{2}}}$ | $\left[ 0,2 \right]$ |
| Camberra | $d_{\mathrm{XY}}=\sum_{j=1}^{n} \frac{\left\vert x_{j}-y_{j} \right\vert}{\left\vert x_{j} \right\vert+\left\vert y_{j} \right\vert}$ | $\left[ 0,n \right]$ |
| Soergel | $d_{\mathrm{XY}}=\frac{\sum_{j=1}^{n} \left\vert x_{j}-y_{j} \right\vert}{\sum_{j=1}^{n} \max\left\{ x_{j},y_{j} \right\}}$ | $\left[ 0,2 \right]$ |
| Lance-Williams/Bray-Curtis I | $d_{\mathrm{XY}}=\frac{\sum_{j=1}^{n} \left\vert x_{j}-y_{j} \right\vert}{\sum_{j=1}^{n} \left( \left\vert x_{j} \right\vert+\left\vert y_{j} \right\vert\right)}$ | $\left[ 0,1 \right]$ |
| Wave-Edges | $d_{\mathrm{XY}}=\sum_{j=1}^{n} \left( 1-\frac{\min\left\{ x_{j},y_{j} \right\}}{\max\left\{ x_{j},y_{j} \right\}} \right)$ | $\left[ 0,2n \right]$ |
